# SPR Analysis of SUMO-Murine Rap1-Interacting Factor 1 C-Terminal Domain Interaction with G4

**DOI:** 10.3390/bios12010037

**Published:** 2022-01-12

**Authors:** Sana Alavi, Hamed Ghadiri, Bahareh Dabirmanesh, Khosro Khajeh

**Affiliations:** 1 Department of Nanobiotechnology, Faculty of Biological Sciences, Tarbiat Modares University, Tehran 14115-154, Iran; sana.alavi@modares.ac.ir; 2 Department of Biochemistry, Faculty of Biological Sciences, Tarbiat Modares University, Tehran 14115-154, Iran; Hamed.ghadir@modares.ac.ir (H.G.); dabirmanesh@modares.ac.ir (B.D.)

**Keywords:** mouse Rap1-interacting factor 1, G-quadruplex, surface plasmon resonance, molecular interaction, dissociation constant (K_D_)

## Abstract

One of the advantages of surface plasmon resonance is its sensitivity and real-time analyses performed by this method. These characteristics allow us to further investigate the interactions of challenging proteins like Rap1-interacting factor 1 (Rif1). Rif1 is a crucial protein responsible for regulating different cellular processes including DNA replication, repair, and transcription. Mammalian Rif1 is yet to be fully characterized, partly because it is predicted to be intrinsically disordered for a large portion of its polypeptide. This protein has recently been the target of research as a potential biomarker in many cancers. Therefore, finding its most potent interacting partner is of utmost importance. Previous studies showed Rif1’s affinity towards structured DNAs and amongst them, T_6_G_24_ was superior. Recent studies have shown mouse Rif1 (muRif1) C-terminal domain’s (CTD) role in binding to G-quadruplexes (G4). There were many concerns in investigating the Rif1 and G4 interaction, which can be minimized using SPR. Therefore, for the first time, we have assessed its binding with G4 at nano-molar concentrations with SPR which seems to be crucial for its binding analyses. Our results indicate that muRif1-CTD has a high affinity for this G4 sequence as it shows a very low K_D_ (6 ± 1 nM).

## 1. Introduction

Bio-sensing platforms can be used for early diagnosis and monitoring of pathological conditions such as cancer diseases e.g., it may significantly improve prognosis and survival rates [1]. Biosensors represent excellent analytical tools for the research on potential biomarker candidates to analyze the two partners’ affinity towards each other. Among the different biosensing techniques, plasmonic platforms can be used to investigate different classes of biomolecules of clinical interest [1]. The SPR technique has been proven to be one of the most versatile frameworks for biosensors applications in different scientific areas. SPR biosensors provide excellent analytical performance owed to their label-free, real-time approach [2].

For each bio-sensing platform, the interacting partners and their affinity are very crucial in determining the specificity and screening of the suitable partner to be embedded. Also, to screen a suitable drug amongst the candidates which can prevent the molecule of interest from functioning or interfering with its normal role. As the relation between Rif1 and human diseases such as cancers and some hereditary diseases is confirmed in recent studies, therefore this protein could be considered as a future biomarker [3,4,5].

Rap1-interacting factor 1 (Rif1) was originally discovered in budding yeast as a protein that interacts with the telomeric DNA-binding protein, Rap1 [6]. It is conserved from yeast to mammals [7,8]. Mammalian Rif1 has diverse roles in DNA repair and replication fork, by accumulating at DNA double-stranded breaks (DSBs) [9,10], and at stalled replication forks [11], respectively. Another key conserved function of Rif1 is the global regulation of replication timing [12,13,14]. It was shown that not only DNA replication, but also other chromosome transactions are regulated by the chromatin architecture generated by Rif1 [11]. It is suggested that chromatin architecture formation by Rif1, is performed through its interaction with G-quadruplex (G4) DNA [15].

In the 2418 amino acid polypeptide of the muRif1, there are two conserved regions reported. The ~1000-amino acid N-terminal domain (NTD) known to constitute HEAT/Armadillo repeats [9] was reported to be required for accumulation of Rif1 at DSB sites [16] and the ~350-amino acid C-terminal domain (CTD) [7] is necessary for Rif1 to interact with phosphoprotein phosphatase 1 (PP1) [8,17,18]. Due to the weak similarity of its CTD to the C-terminal domain of RNA polymerase α-subunit, it was assumed that this region is a DNA-binding domain [7]. It was recently reported that both N-terminal and C-terminal domains individually bind to G4, although both domains are required for high-affinity binding to G4 [19].

Previously it was reported that Rif1 from fission yeast (*Shizosaccharomyces pombe*, SpRif1) binds G4 with high specificity and affinity [15], and a recent study showed that the purified murine Rif1 is also capable of interacting with G4 [19]. Its specificity for G4 structures suggests that structure-specific DNA binding plays a conserved and important role in Rif1 function [20]. G4 structures have been associated with replication origins and it was reported that they are important for origin firing in the model replication origins in DT40 cells [21,22].

Many studies are ongoing to investigate the most effective partner of Rif1 which, varies from cruciform to G-quadruplexes [15,19,20,23]. Recent studies are enforcing the latter molecule to be binding Rif1 with higher affinity [15,19,20,23,24]. However, all the analyses conducted until now have been by the gel shift method. Therefore, we are investigating the G4-interacting property of Rif1 with its most potent reported partner, i.e., T_6_G_24_ [15,19,23] by a more sensitive method of SPR.

Here, we have expressed muRif1-CTD in *E. coli* and tried to increase the solubility of the recombinant protein using the SUMO-fused tag. It was also reported that Rif1 itself is SUMOylated specifically in the G phase of the cell cycle [25]. The functionality of the purified protein, i.e., its interaction with G4 was examined through gel shift assays. Furthermore, we designed an SPR based platform, to quantitatively analyze the interaction between SUMO-muRif1-CTD and G4.

## 2. Materials and Methods

### 2.1. MuRif1-CTD Expression, Solubility Testing

Both pPal vector (Bio-Rad, USA) and pETite™ N-His SUMO vector (Lucigen Corporation, Middleton, WI) were implemented for expressing muRif1-CTD which respectively add profinity eXact and SUMO tags at the N-terminal of the protein.

Strategies examined in the solubilization of muRif1-CTD protein were categorized in three major groups depending on the factors changed or added during the induction and lysis stage. These groups were:

- Different buffers and additives were explored to obtain the best condition for cell lysis;
- Diverse additives were incorporated during the expression of muRif1-CTD to the culture medium after induction by IPTG;
- Various induction methods.

Protein expression and solubility examination were conducted through SDS-PAGE analyses by the Laemmli method [26]. For this purpose, bacterial culture was harvested after induction and the bacterial pellet was sonicated seven times (7 s on, 30 s off) in the lysis buffer in each condition. The lysate was centrifuged at 12,000× *g* for 20 min and the supernatant was collected for the solubility analyses. The pellet after centrifugation was also examined to assure that the protein was expressed, and it was in its insoluble form, if not present in the supernatant fraction.

### 2.2. Protein Purification and Tag Removal Analyses

Luria Bertani (LB) medium containing 30 μg/mL kanamycin was inoculated with a selected positive colony of BL21 containing the desired gene. The culture was grown overnight at 37 °C and shaken at 220 rpm. One hundred milliliters of LB medium was inoculated by 1% of the pre-culture. After reaching OD_600nm_ 0.7–0.9, IPTG was added to a final concentration of 1 mM. Shaking was continued at 37 °C for 3 h. The culture was harvested at 4000× *g* for 15 min. The pellet was resuspended in the lysis buffer (Tris-HCl 50 mM, 1 M NaCl, pH 7.4, 20 mM Imidazole). Sonication (7 s on, 30 s off) was repeated seven times. The lysate was centrifuged at 12,000× *g* for 20 min and the supernatant was collected for the purification step.

The SUMO-muRif1-CTD construct has 6× His tags at the N-terminal, and it was purified through immobilized metal affinity chromatography (IMAC) with nickel NTA (nickel agarose) resin (Qiagen Hilden, Germany). Purification was performed by equilibrating the column with the same buffer as the lysis buffer, i.e., Tris-HCl 50 mM, 1 M NaCl pH 7.5, 20 mM imidazole with 1 mL/min flow rate. The protein of interest was stripped off the column with 250 mM of imidazole. For SPR analyses, NaCl salt was replaced with KCl. All fractions were collected and analyzed by SDS-PAGE.

Using Amicon-10 kDa or Micron-10 kDa (Millipore, Billerica, MA, USA), purification buffer was exchanged with the cleavage buffer condition for SUMO Expresso^®^ protease (Lucigen, Middleton, WI, USA), i.e., 20 mM Tris-HCl pH 8.0, 150 mM NaCl, 10% glycerol to cleave the tag (for stability reasons, we were forced to increase the salt to 500 mM NaCl). With the addition of fresh dithiothreitol (DTT) to a final concentration of 2 mM in the exchanged buffer, the cleavage condition was obtained. One unit of protease per 10–100 μg of the fusion protein was added. The mixture was incubated at 30 °C for 1 h, or at 4 °C overnight (both conditions were analyzed). The cleavage reaction was loaded on the nickel NTA column once again to remove the SUMO tag, any remaining un-cleaved fusion protein, and the protease itself which was His-tagged. Target protein in the flow-through was collected for further investigation.

### 2.3. G4 Formation Investigations

Synthesized sequence capable of G4 formation was dissolved in water for injection (WFI) and stored at −20 °C to a final concentration of 100 µM. G-quadruplex structures generation was performed by heat denaturing–renaturing treatment. The putative G4 structure sequence was heat-denatured at 90 °C for 5 min at 4 μM concentration and then diluted by 20 mM Tris-HCl pH 7.5 and 100 mM KCl to 1 μM and gradually cooled to room temperature over 2 h [15,27,28]. G4 formation with the same procedure in the presence of 100 mM MgCl_2_ was also examined.

G-quadruplex structure formation detection was carried out through 3,6-Dimethyl-2-(4-dimethylaminophenyl) benzothiazolium cation (ThT) fluorescence [27,28] and Circular Dichroism (CD) spectroscopy [29] was used to assess the type of G4 formed.

The concentration of ThT was calculated using the molar extinction coefficient of 36,000 M^−1^ cm^−^^1^ in water at 412 nm and a stock of 5 mM was prepared. A mixture of ThT and oligonucleotide at final concentrations of 1 and 0.5 μM was prepared at room temperature. The 1:1 highly fluorescent complexes were predominant having fluorescence emission maximum at 490 nm after excitation at 425 nm [27] in the fluorescence spectrophotometer (LS 55, Perkin Elmer Co., Waltham, MA, USA). Fluorescence scans were performed every 2 nm between 450 and 700 nm under excitation at 425 nm.

CD spectra were recorded on Jasco J-715 spectropolarimeter (Tokyo, Japan) using a 1-cm path length quartz cuvette in a reaction volume of 300 μL at 20 °C. Scans from 220 to 320 nm were performed with a rate of 200 nm/min, 1 nm pitch, and 1 nm bandwidth. The DNA concentration was 5 μM. For each experiment, an average of two scans was taken, the spectrum of the buffer with 20 mM Tris-HCl and 100 mM KCl was subtracted, and the data were zero-corrected at 320 nm. This experiment was repeated with HEPES buffer in place of Tris-HCl as this buffer was used as the binding buffer for our further analyses.

### 2.4. Electrophoretic Mobility Shift Assay (EMSA) Set Up for G4/muRif1-CTD Interaction Detection

Setting up the EMSA assays was conducted through optimizing different polyacrylamide gel electrophoresis (PAGE) conditions and visualization methods (data not shown here). The procedure for execution of EMSA was that muRif1-CTD and G4 were incubated in the binding buffer at 30 °C for 30 min. The whole reaction was loaded onto PAGE, and the gel was run at 60 V for 2 h in 1× TBE (different other running buffers had also been tested, including; 1× TBE + 10% Glycerol + 50 mM KCl and 1× TBE + 50 mM KCl). SUMO-muRif1-CTD was incubated with G4 in the same condition. Furthermore, another protein with a molecular mass of 35 kDa which was previously incorporated into the same vector in our lab, was used as a control to assess whether the SUMO part of the protein is interacting with the G4 structure or this interaction is nonspecific. This experiment was performed with the same conditions as for muRif1-CTD and SUMO-muRif1-CTD.

### 2.5. Interaction Analyses by SPR

MuRif1-CTD and G4 interaction analyses were performed using the Xantec SR7500 instrument (Germany), equipped with an automatic flow injection system. The instrument detects changes in the refractive index as the micro-refractive index (µRIU), which is proportional to the quantity (mass) of analyte interacting with the surface. The streptavidin pre-treated sensorchip surface was washed with 60 s aliquots of 50 mM NaOH to remove loosely retained streptavidin. Biotinylated G4 structures at 5′-end previously formed (please refer to section *G4 formation investigations*) and confirmed by CD analyses were immobilized on a chip and used as bait in SPR analysis. Streptavidin–Biotin coupling was conducted at 25 nM concentration of G4 at 20 μL/min only on one flow cell. The capturing level was set to 100 μRIU. The unbound ligand was washed away through consecutive injections of 1 M KCl in running buffer. Running buffer was 20 mM HEPES pH 7.4, 500 mM KCl with 0.005% (*w*/*v*) Tween 20, and the chip surface was maintained at 25 °C (note: the same running buffer with lower KCl concentration and without detergent was also tested).

A key factor in determining the kinetic constants by the SPR method is that the free protein concentration in the matrix should quickly equilibrate with the flow solution. The binding reaction is considered to be mass transfer limited if the association reaction is much faster than mass transport. Therefore, the interaction kinetics is considered to be true if the transport is fast and the association is slow. Therefore, the mass transport rate is a critical factor that must be considered. To minimize the mass transport limitation, it is recommended to use a higher flow rate (≥50 μL/min) and low surface densities of the immobilized ligand.

The interaction between G4-DNA (as a ligand) and the purified SUMO-muRif1-CTD was analyzed at four different concentrations at 100 μL/min flow rate. The association phase was followed up for 60 s, while the dissociation phase was further observed for at least 120 s. The experiment conditions were optimized and the resultant sensorgrams for each interaction were analyzed using the Scrubber^®^ analysis program (BioLogic Software Pty. Ltd., Canberra, Australia) according to the 1:1 Langmuir ligand model and kinetic parameters were estimated by fitting the data using the same program. The specificity of the biosensor was evaluated by using another SUMO-tagged protein with approximately the same molecular weight partner protein (Uricase) as muRif1-CTD as a control. Moreover, in the process of each analysis, a few buffer injections without any analyte were used as a blank.

## 3. Results

### 3.1. Prokaryotic Expression and Purification of SUMO-muRif1-CTD

The muRif1-CTD was successfully cloned into the prokaryotic vector containing the profinity eXact™ or SUMO tag at its N-terminal. This was confirmed with the double digestion and sequencing of the inserted gene amplified by colony PCR.

Different methods have been utilized to solubilize muRif1-CTD. Table 1 summarizes all the measures attempted and the result achieved from each action. Both supernatant and pellet after sonication of bacteria expressing the muRif1-CTD were analyzed through SDS-PAGE. Overall, among all the methods tried out, other than incorporating the sarcosyl detergent, urea, and SUMO solubilizing tag, none yielded any significant increase in the solubility of this protein.

**Figure 1:**
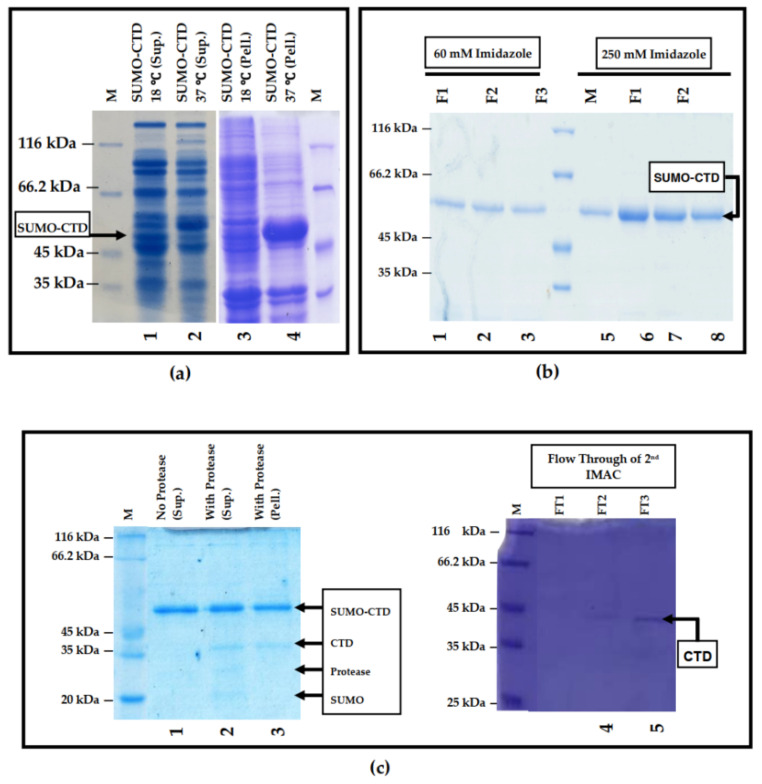
SUMO-muRif1-CTD expression and purification analyses. (**a**) Analyses of SUMO-muRif1-CTD expression in *E. coli* BL21 (DE3): Expression condition was T.B medium, 1 mM IPTG in 37 °C and 18 °C for 24 h. Samples were subjected to SDS-PAGE and stained with colloidal Coomassie Brilliant Blue. M: Marker; lane 1: Supernatant of SUMO-muRif1-CTD harboring bacterium expressed in 18 ℃ induced with IPTG after lysis; lane 2: Supernatant of SUMO-muRif1-CTD harboring bacterium expressed in 37 °C induced with IPTG after lysis; lane 3: Pellet of SUMO-muRif1-CTD harboring bacterium expressed in 18 °C with IPTG after lysis; lane 4: Pellet of SUMO-muRif1-CTD harboring bacterium expressed in 37 °C with IPTG after lysis. (**b**) SDS-PAGE analysis with colloidal Coomassie Brilliant Blue staining for purification of SUMO-muRif1-CTD by nickel NTA affinity chromatography. Purification was performed with the same lysis buffer in which bacterial cell was lysed (Tris-HCl 50 mM, 300 mM NaCl, 20 mM imidazole) and eluted with different imidazole concentrations. M: Marker; lane 1–3: Elution fractions 60 mM of imidazole; lane 5–8: Elution fractions 250 mM of imidazole. The arrow indicates the position of the recombinant His-tagged SUMO-muRif1-CTD (41 kDa), which migrates at approximately 55 kDa. (**c**) Cleavage of CTD-SUMO by Lucigen SUMO Expresso^®^ protease. Cleavage condition was Tris-HCl pH 8, 150 mM NaCl, 1 mM DTT and 10% glycerol. M: Marker; lane 1: Cleavage reaction without protease (supernatant); lane 2: Cleavage reaction with protease (supernatant); lane 3: Cleavage reaction with protease (pellet); lane 4 and 5: Purified muRif1-CTD after cleavage.

Expression of SUMO tagged protein was conducted at 37 °C for 3 h in terrific broth (TB) medium under 220 rpm agitation. These conditions resulted in the protein being in its insoluble form (Figure 1a). To resolve this issue, the expression temperature was decreased to 18 °C for 20 h (Figure 1a). The amount of IPTG for expression remained at 0.5 mM. With these conditions, a fairly good amount of SUMO-muRif1-CTD protein was obtained in the soluble portion of the cell lysate (supernatant), and this amount seemed to be sufficient to further experiment with this protein.

SUMO-muRif1-CTD eluted from IMAC and fractions containing purified protein were analyzed by SDS-PAGE. Although the protein could be eluted by 60 mM imidazole, 250 mM imidazole was used for the elution step to obtain more concentrated fractions (Figure 1b). The protein was ~99% pure based on the SDS-PAGE analysis (Figure 1b). Theoretically, SUMO-tagged muRif1-CTD has a molecular weight of ~41 kDa, but it migrates at ~50 kDa in the gel (Figure 1b). This is not only because of the SUMO tag which migrates slower in the gel [30], but also the CTD movement itself that has been reported to be retarded in the gel [19].

The results of the cleavage with His-tagged Expresso^®^ SUMO protease suggested that after 30 min or overnight incubation of the protease nearly 50% of the protein was in its insoluble form. Using the second step of nickel affinity chromatography, protease, SUMO tag, and other small impurities present in the cleavage reaction were removed and cleaved protein, i.e., SUMO less muRif1-CTD remained in the flow-through (Figure 1c). The Purity of muRif1-CTD was analyzed by SDS-PAGE. Theoretically, muRif1-CTD protein has a molecular mass of 29 kDa but this protein migrated at 35 kDa in the gel.

### 3.2. G4 Formation Investigation

**Figure 2:**
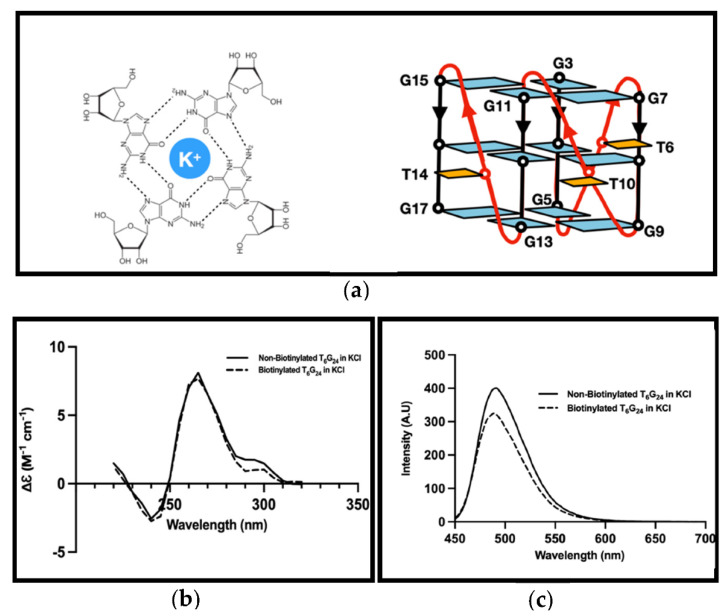
G4 formation analysis. (**a**) G-Quadruplex structure (left) and schematic representation of main folding topology formed by the G4 structure used in this experiment (T_6_G_24_) in vitro (right). (**b**) CD analysis of non-biotinylated (solid lines) and biotinylated (dashed lines) T_6_G_24_ sequence formed in the presence of KCl. (**c**) Fluorescence analysis of non-biotinylated (solid lines) and biotinylated (dashed lines) T_6_G_24_ sequence formed in the presence of KCl.

It was expected for the T_6_G_24_ sequence to form a parallel G4 structure (Figure 2a). Results for G4 formation analyses of the biotinylated nucleotide sequence are presented in (Figure 2b,c). Biotinylation did not affect the G4 formation, and in the presence of this tag, the G4 structure was formed.

A characteristic circular dichroism (CD) spectral profile with a positive band at around 260 nm and a negative band near 240 nm was observed in the CD spectrum of the G4 sequence (T_6_G_24_) showing a parallel G4 structure that is usually formed in the presence of K^+^ for the sequence (Figure 2b).

G4 formation analyses of the T_6_G_24_ sequence by fluorescence analyses using the ThT method showed an increase in the 490 nm emission of the mixture containing ThT and G4 formation (Figure 2c). The G4 sequence with 4 μM concentration was used for these analyses. Moreover, using a higher concentration of G4 resulted in higher fluorescence emittance.

It was revealed that the peak pertaining to G4 formation was not seen for the G4 formed in the presence of MgCl_2_. Using MgCl_2_ did not aid in creating any topology for the G4 sequence (data not presented) as Mg^2+^ seems to destabilize the formation of G4 structures. Moreover, the G4 structures formed by this sequence may be composed of both inter and intra-molecular G-quadruplexes. G4 formation curves were generated using GraphPad Prism 5.0 (GraphPad Software, Inc., San Diego, CA, USA).

### 3.3. Surface Plasmon Resonance Investigations

**Figure 3:**
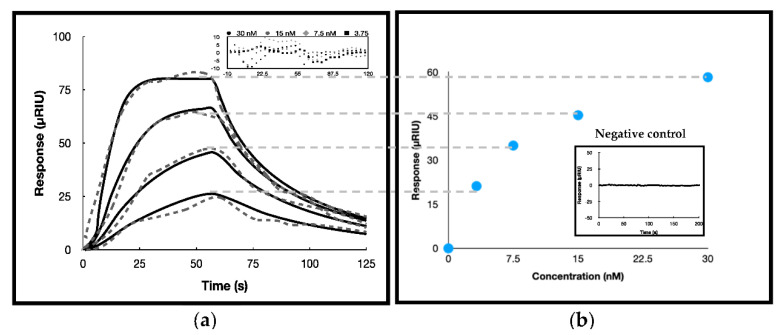
(**a**) Binding kinetic fitting of experimental SPR sensorgrams (grey dashed lines) with corresponding Langmuir 1:1 ligand model simulation (black solid lines) for G4 and SUMO-muRif1-CTD interaction. Running buffer was 20 mM HEPES pH 7.4, 500 mM KCl, the association time was 60 s and flow rate in all the analyses was 100 µL/min. SUMO-muRif1-CTD concentration was 3.75, 7.5, 15 and 30 from bottom to top. The inset indicates the residual plots for these analyses. (**b**) Steady-state fitting of the sensorgrams. The inset indicates the difference sensorgram of the control protein injected for checking the specificity of the sensorchip prepared.

To further kinetically analyze the affinity of T_6_G_24_ with SUMO-muRif1-CTD, SPR analysis was performed with one flow cell unmodified as a reference and the other flow cell with immobilization of the G4 structures (sensing channel) to achieve equilibrium binding constant K_A_ from the direct measurements of complex association (k_a_) and dissociation (k_d_) rates in real time. Biotinylated formed G4 structures were successfully immobilized on the sensorchip pre-coated with streptavidin till 100 μRIU. Different concentrations of purified SUMO-muRif1-CTD (3.75, 7.5, 15, 30 nM) diluted in running buffer injected showed that the response was increased using higher concentrations of SUMO-muRif1-CTD (Figure 3a). The response obtained is from deducing the signal of the sensing channel from the reference channel. It was revealed that reducing the KCl concentration caused the multimerization/aggregation of SUMO-muRif1-CTD, and to maintain the status of the protein, KCl had to be present at least at 500 mM concentration and be freshly prepared. On the other hand, it was shown that the interaction did not occur in the presence of higher concentrations of KCl (750 mM and 1000 mM) in the sample buffer while maintaining 500 mM KCl in the sample and running buffer stabilized both SUMO-muRif1-CTD protein and the complex formation. Analyses of multimerized/aggregated SUMO-muRif1-CTD in 50 mM KCl displayed higher maximum response (R_max_) in comparison to responses achieved from the so-called dimeric form.

Moreover, injection of a control protein with nearly the same molecular mass attached to the SUMO tag showed that this protein was unable to interact and the sensorgram did not show any difference in the channels’ sensorgrams detected (Figure 3b).

By fitting the difference sensorgrams (i.e., the response subtracted from the reference to remove the bulk refractive index) using a 1:1 binding model, the kinetic data were calculated. The data were fitted on both a mass transfer limited model (k_m_ (3.0 ± 0.2)10^8^) and Langmuir model where no mass transfer constant was included. As the KD reported by two models were very close to each other we report the data where no mass transfer constant is incorporated. The data for SUMO-muRif1-CTD and G4 interaction in 500 mM KCl concentration were only fitted and the two other conditions could not be fitted by the software. The k_m_ (m: mass transfer) value was calculated to be higher than the k_a_ which shows that the mass transfer limitation did not affect the k_a_ value and the association of SUMO-muRif1-CTD to G4. Moreover, the results of the equilibrium and kinetic analyses revealed approximately the same K_D_ value (within their range of errors) which again confirms that the binding condition was not limited by mass transfer (Table 2).

The K_D_ value obtained from kinetic evaluation was used for calculating the thermodynamic parameter, ΔG°_binding_, using the equation:

ΔG°_binding_ = RT lnK_D_,

where R is the universal gas constant, K_D_ is the dissociation constant, T is the temperature in Kelvin, and ΔG°_binding_ is the changes in Gibb’s free energy due to the binding of muRif1-CTD to immobilized G4. Effective binding free energy of −11.123 kcal/mol (−46.496 kJ/mol) was determined for the G4 and SUMO-muRif1-CTD SPR binding analyses kinetic data. Table 2 reports kinetic and thermodynamic parameters.

## 4. Discussion

Being a crucial protein in DNA replication and repair [11,31], Rif1’s molecular functions need to be dissected in order to understand how exactly this protein regulates many cellular processes. Previously, Rif1 and its different domains were expressed in a mammalian expression system using 293T cells in a convenient and affordable method [19]. The main issue with expressing this protein is that it is predicted to be largely unstructured [20], and the purification of full-length Rif1 or its domains is very difficult [19]. Furthermore, the amount of protein expressed in the native source was very less, and its purification to sufficient quantities was not achieved totally as this protein is associated with the nuclear lamina.

There are many investigations on structured DNAs which interact with the DNA-binding domain of Rif1 including, forked DNA, flap DNA, and cruciform structures [7,15,20]. Competition assays executed by other researchers revealed that the G_24_ DNA sequence—which is known to form a parallel-type G-quadruplex structure—is bound to Rif1 with higher affinity than other structured DNAs [15,23]. Moriyama et al. used the T_6_G_24_ sequence for the EMSA assays and mentioned that the G4 concentration used in their study would be too low to determine convincing K_D_ values for truncated muRif1 proteins, and they reperformed the tests with higher probe (G4) concentrations which resulted in K_D_ values of 33, 61.9, and 18.1 nM for, respectively, 8.33, 16.7 and 38.9 nM probe concentrations [19].

Overall, the considerations to be cautious in Rif1 and G4 interaction studies include; Rif1 protein instability when expressed in prokaryotes [7], Rif1 low expression level in the eukaryotic host [19], G4 oligomerization and its effect on Rif1 binding to G4 [19], and Rif1 domains oligomerization and its impact on the interaction [15,19,23]. For the above-mentioned reasons, we propose the benefits of SPR in real-time and would be able to investigate the interaction and perceive more on what is occurring by online monitoring.

In this study, we attempted to overcome the concerns mentioned above and have implemented the advantages of the SPR method to analyze the interaction of the muRif1-CTD domain with the T_6_G_24_ sequence. We present a new strategy for expressing muRif1-CTD in its soluble form in *E. coli*. We aimed to overcome or minimize the issue presented by Xu et al. for muRif1-CTD expressed in prokaryotes using the maltose-binding protein (MBP) tag which is self-associated [7]. As we also used MBP (data not yet published), we confirm that the SUMO tag seems to be better in maintaining muRif1-CTD in its soluble and stable form.

Moreover, the preliminary investigations for muRif1-CTD interaction with G4 both with and without the tag were performed by EMSA assays. The analysis of both purified muRif1-CTD and SUMO-muRif1-CTD revealed that they could bind to the G4 structure, but there seems to be no interaction detected for muRif1-CTD in the presence of non-G4 structures. To confirm that the experimental conditions were not faulty and had given a false positive, we have used a control protein with nearly the same molecular mass attached to the SUMO tag and investigated its interaction with G4. This protein was unable to interact, and the band for G4 was clearly visible after the binding reaction was completed. Therefore, it was revealed that G4 interacts with Rif1, not the SUMO tag.

Our SPR analyses show that, like many other DNA protein interactions, this interaction was mass transfer limited too, and we have taken the required measures, i.e., using lower ligand concentration in capturing phase, increasing the flow rate to overcome this phenomenon. Additionally, in our SPR analyses, it seemed that SUMO-muRif1-CTD could interact with each other on the chip surface (data not presented here). In this study, reducing the salt concentration yielded higher signal values, and it is suggested that maybe the salt concentration is one of the decisive factors for protein oligomerization/aggregation status.

Our study confirmed the previous studies stating the high affinity of mammalian Rif1-CTD for parallel T_6_G_24_ G4 structure [15,19,20,23]. These results were quantitatively confirmed by a more accurate approach such as SPR. This interaction was salt-dependent, and higher concentrations of salt inhibited the interaction. Salt concentration in the running buffer was important in the dissociation phase as reducing the salt decreased the dissociation rate. Therefore, the results presented here were specific to the SPR condition stated. This result was lower than other studies with the same G4 structure for K_D_ calculation by the gel shift assay as the study was performed by a more precise method [19,24].

Moreover, we have studied the muRif1-CTD (analyte) and G4 (ligand) interaction via the SPR method, which is very accurate and globally accepted for k_a_ and k_d_ reporting. These kinetic values are important not only for understanding the action mechanism of this protein in interaction with its partner, but also to further proceed with the screening of drug candidates. The rates of drug−target complex formation (k_a_) and breakdown (k_d_) are important in drug discovery studies which is one of our reasons for investigating the kinetics of this binding. Since k_a_ and k_d_ depend on the difference in free energy between the ground and transition states of the binding reaction coordinate, efforts to improve drug potency may have unpredictable effects on the kinetics of drug–target complex formation and breakdown.

SPR analyses conducted here were for SUMO-tagged muRif1-CTD. One of the advantages of the SUMO tag—either as a fusion tag or as a cellular modification—is that it will mostly not have any adverse impact on the conformation of the proteins [32,33,34,35]. It was shown that SUMOylation of some repair-associated cellular proteins such as Rad1 [36] and Rad52 [37] decreased their DNA-binding affinity and facilitated their dissociation from DNA. In other studies, it was shown that SUMOylation of Yku70 and Oct4, enhances its DNA-binding affinity [38,39]. It would be interesting to analyze the possible effect of SUMOylation on conformational changes in Rif1, in our future studies.

## 5. Conclusions

In summary, designing and setting up a platform for the expression of Rif1 or each of its domains is very important in achieving suitable amounts of Rif1 protein for the structural analysis of this protein. At the same time, analysis of Rif1 interaction with G4 DNA and other cellular proteins quantitatively can create a suitable basis for future practical studies such as drug discovery or biomarker investigation studies.

## Figures and Tables

**Table 1:** MuRif1-CTD solubilization strategies and their effect.

Strategy	Agent Used	Result
Buffer type and pH	Tris and Phosphate 25 mM pH range: 6–8.	Insoluble
Lysis buffer Additives	Salts: NaCl (100 mM), KCl (100 mM), ammonium sulfate (100 mM and 500 mM), sodium acetate (100 mM).	Insoluble
	Urea (0.1, 0.5 M) ^1^	
	Amino acids: Arg (375 mM)**,** Arg (1 M), Glu (5 mM), Asp (50 mM), Glycine-betaine (10, 100 and 1000 mM), Arg+Gly (each 50 mM).	Insoluble
	Reducing Agents: 2-ME (0.05%), DTT (5 mM).	Insoluble
	Detergents: Triton X-100 (0.2% and 1% (*v*/*v*)), SDS (0.1% (*w*/*v*)), Sarcosyl (0.1–0.3% (*w*/*v*)) ^2^	Insoluble
	Fatty Acid: Glycerol (5 and 10% (*v*/*v*)).	Insoluble
	Sugars: Trehalose (0.5 M), sorbitol (0.5 M), sorbitol + trehalose+Arg (each 50 mM).	Insoluble
Induction condition	Culture media (TB, LB).	Insoluble
	Time of induction (3, 6, and 18 h) for MuRif1-CTD 270 aa and 221 aa.	Insoluble
	Temperatures (18 °C, 25 °C, 37 °C), and heat shock.	Insoluble
	Effect of temperature and time of induction combination. Effect of medium, temperature and time of expression combination.	Insoluble
	Different IPTG concentrations (0.1, 0.25, 0.5, 1, and 2 mM). Different IPTG concentrations (0.01, 0.1, 0.5 mM) and time of induction (12, 20, and 24 h).	Insoluble
	Induction methods (Lactose in place of IPTG).	Insoluble
	OD for induction (0.8, 1, and 1.2) and glucose addition.	Insoluble
	Hosts (BL21, BL21 (DE3), Rosetta gami, BL21 (DE3) pLyse) with different time of induction.	Insoluble
Induction additives	Amino acid: Arg (0.5 M).	Insoluble
	Alcohol: Ethanol (3% *v*/*v*).	Insoluble
	Sugar: Trehalose (0.5 M), sorbitol (0.5 M) and a mixture of trehalose, sorbitol and Arginine, Betain (100 mM)	Insoluble
Solubilization tag	SUMO	Soluble

^1^ Urea at higher concentrations of 2 M above was able to solubilize the protein but the refolding was not successful and the protein was not stabilized well. ^2^ Sarcosyl was able to solubilize the protein at concentrations above 0.2% *w/v* but the detergent was attached to the structure.

**Table 2:** Binding kinetics and thermodynamic parameters of G4/muRif1-CTD from SPR analysis.

k_a_(M^−1^ s^−1^)	k_d_ (s^−1^)	K_D_ (nM)		R_max_^1^	Response Standard Deviation	ΔG°_bind_^2^(kcal/mol) *
		Kinetic Fit	Steady-State Fit			
(4.2 ± 0.2) 10^6^	0.030 ± 0.001	7.1 ± 0.4	6 ± 1	99 ± 2	4.856	−11.123 ± 0.05

^1^ The average of the squared differences between the measured data points and the corresponding fitted values. ^2^ ΔG°_bind_ = RT lnK_D_. * kcal/mol = 4.184 kJ/mol.
